# High spatiotemporal resolved imaging of ultrafast control of nondiffracting surface plasmon polaritons

**DOI:** 10.1515/nanoph-2023-0074

**Published:** 2023-05-16

**Authors:** Hanmin Hu, Boyu Ji, Lun Wang, Peng Lang, Yang Xu, Zhenlong Zhao, Xiaowei Song, Jingquan Lin

**Affiliations:** School of Physics, Changchun University of Science and Technology, Changchun 130022, China; Zhongshan Institute of Changchun University of Science and Technology, Zhongshan 528400, China; College of Electrical and Information Engineering, Quzhou University, Quzhou 324000, China

**Keywords:** high spatiotemporal resolved imaging, nondiffracting surface plasmon polaritons, photoemission electron microscope, ultrafast spatiotemporal control

## Abstract

Nondiffracting Bessel surface plasmon polariton (SPP) beams, which have unique self-healing, non-divergence, and linear transmission properties, have charming applications in plasmonic devices and on-chip interconnection circuits. Here we first realize, to the best of our knowledge, the ultrafast control and imaging of the Bessel SPP pulse on the nano-femto scale in the experiment. We demonstrate ultrafast control of Bessel SPP pulse switching by controlling the instantaneous polarization state of the excitation light. Moreover, this variation process is directly mapped on the nano-femto scale by time-resolved two-color photoemission electron microscopy. The results are well reproduced by the finite-difference time-domain (FDTD) method. The current study of ultrafast control and spatiotemporally imaging the switching process establishes an experimental paradigm for revealing the complex mechanisms in ultrafast control of nondiffracting SPP and are useful for developing high-speed, highly-integrated nanophotonic devices, and on-chip circuits.

## 1 Introduction

Surface plasmon polaritons (SPP) exhibit intriguing properties of strong field enhancement and subwavelength field confinement [1], which makes it promising in applications ranging from micromanipulation [2, 3], optical sensing [4], sub-wavelength imaging [5], and fixed-point Raman enhancement [6, 7]. More importantly, SPP, as propagating bound oscillations of electrons at the dielectric/metal interface, has the ability to break the limit in the speed of nanoscale electronics and the size of terahertz dielectric photonics, which makes it play an essential role in an information carrier in on-chip interconnection circuits and highly-integrated nanophotonic devices [8, 9]. Thus, complementary efforts focus on the construction and development of various functional plasmonic devices, such as waveguides [10], beam splitters [11], multiplexers [11–13], switches [14, 15], and logic gates [16], etc.

Nevertheless, the inherent diffraction nature of SPP and the limitation of metal absorption and surface roughness scattering on SPP transmission will hinder the high integration of plasmonic devices and induce signal crosstalk in practical applications [17, 18]. Inspired by the nondiffracting light beams concept in three-dimensional space [19], the two-dimensional nondiffracting Bessel SPP beams with properties of self-healing and non-divergence are free from the above issues [17, 20], [21], [22], [23], [24], [25], [26]. Meanwhile, the unique straight transmission trajectory of the Bessel SPP beam makes it more advantageous for information transmission in on-chip interconnection circuits and integrated combinations between multiple devices than other types of nondiffracting SPP beams [25, 27].

The manipulation of SPP is a key step for the realization of plasmonic devices. The research on the manipulation of nondiffracting SPP beams has gradually developed from the early static passive space manipulation scheme [22, 24, 27] to the dynamic active space manipulation scheme [20, 21, 23, 25, 28, 29] and recently it is an emerging trend that active ultrafast manipulation of nondiffracting SPP pulses meets the demands for ultrafast information transmission and processing in applications such as on-chip interconnection circuits and plasmonic devices [30, 31]. Accordingly, the nano-femto scale investigation of ultrafast control of Bessel SPP pulses is vital [32, 33], however, it is still lacking. In addition, owing to the short transmission distance of nondiffracting SPPs excited by miniaturized structures, a direct characterization near the device is required. Photoemission electron microscopy (PEEM) has exhibited unprecedented power in the characterization of SPP due to the fact that it uniquely combines the requisite spatial and temporal resolution and is ideally suitable for imaging the dynamical evolution of the plasmonic field [34]. However, the traditional pump-probe characterization scheme based on monochromatic PEEM will be hindered by the static interference fringe entanglement, and it is difficult to directly characterize the spatiotemporal evolution of SPPs beam near the device [11, 35]. Therefore, a simple and effective experimental method for ultrafast manipulation of nondiffracting SPP pulses and the utter imaging of the corresponding spatiotemporal evolution process is urgently needed.

In this paper, to the best of our knowledge, we first achieve control of Bessel SPP pulses on the nano-femtosecond scale in the experiment. The ultrafast control of Bessel SPP pulses on the femtosecond time scale is realized by changing the instantaneous polarization state via adjusting the time delay of the two orthogonally polarized incident light pulses in the composite catenary structure. The experiment results are well reproduced by finite-difference time-domain (FDTD) simulations. Moreover, we directly mapped this ultrafast switching process by time-resolved two-color PEEM. Different from previous monochromatic pump-probe detection schemes, our proposed three-beam two-color PEEM detection scheme enables the overlapping of the pump and probe pulses which is an advantage for complete imaging of the spatio-temporal manipulation of nondiffracting SPPs. We believe that our work provides a new path for manipulation and ultrahigh spatiotemporal mapping of nondiffracting SPP pulse, which can be applied to construct and characterize ultrafast information processing systems as well as ultrafast switches in plasmonic nanocircuits.

## 2 Results and discussion

**Figure 1:**
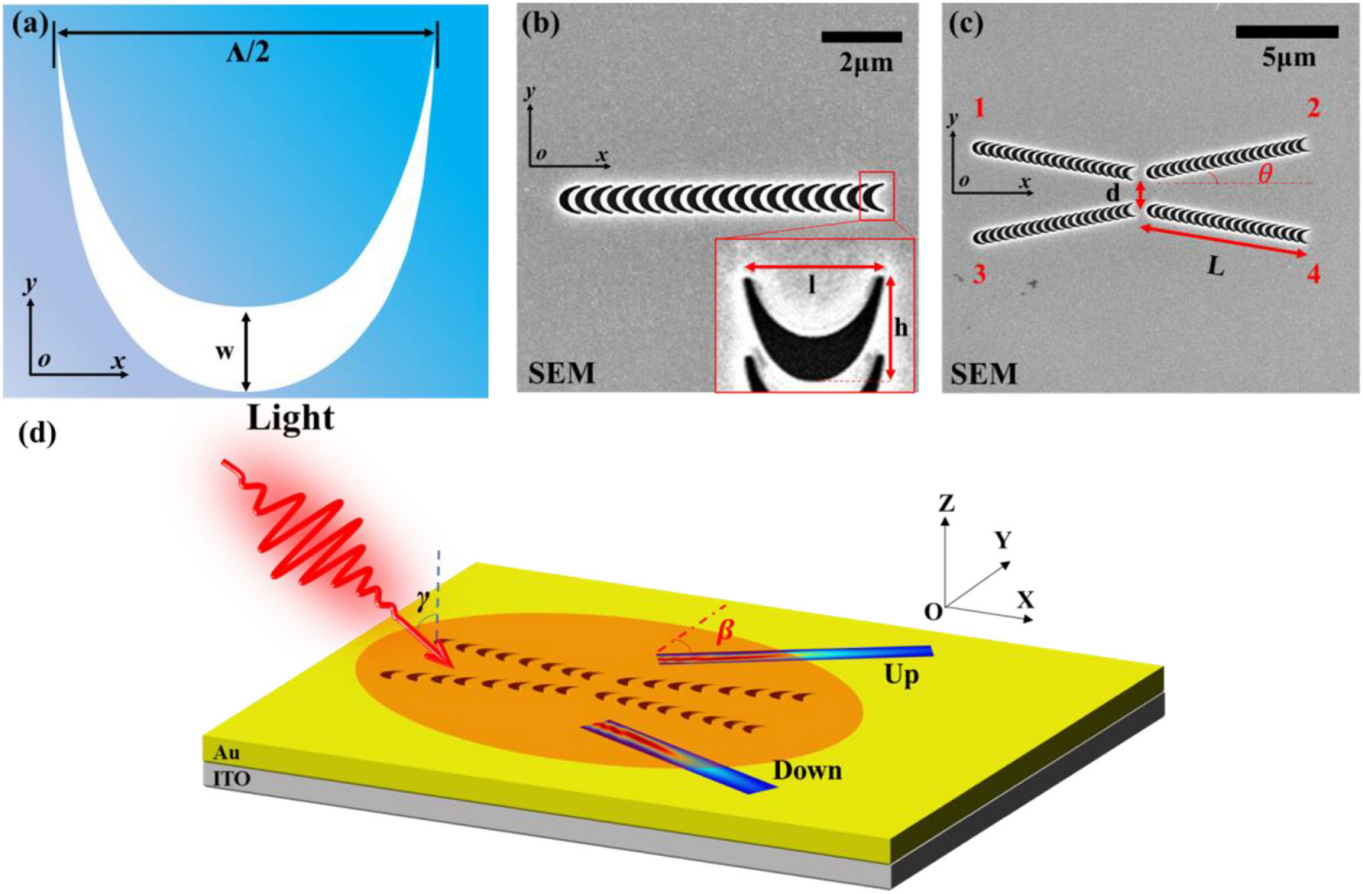
The schematic of the composite catenary structure. (a) The schematic illustration of catenary apertures etched on the gold film. (b) The schematic of the scanning electron microscopy (SEM) image of the single catenary aperture array. The inset is the magnification image of the local structures. (c) The schematic of the SEM image of the composite catenary structure includes four catenary aperture arrays with a tilt of 10°. (d) Schematic diagram of the Bessel SPP beams excited by the composite catenary structure under oblique incidence. The main lobe of the excited Bessel SPP beams will be launched from up and down the structure along different propagation paths.

The catenary curve has been used in optics [36, 37] and plasmonics [38] recently. To realize the active manipulation of Bessel SPP beams, we designed the composite structure based on the catenary curve. Figure 1a depicts the catenary aperture obtained by shifting the catenary curve with *w*. The catenary curve can be written as follows:

(1) $$y=-\left(\Lambda /2\pi \right)\ln\left|\cos\left(2\pi x/\Lambda \right)\right|$$

where Λ is a constant which determines the lateral size of a single catenary curve. To realize the effective excitation and active polarization control of SPP under 65° oblique incident 750 nm light illumination. A single catenary aperture element corresponds to the parameters of a vertical displacement of *w* = 250 nm and the catenary constant value Λ = 1600 nm. The height of a single catenary element is *h* = 550 nm, and its lateral width is *l* = 742 nm (less than the excitation wavelength of 750 nm). Considering that the endpoints on the left and right sides in Equation (1) will approach infinity, the catenary aperture element was truncated at the two ends in the practical design and fabricating process, and the SEM images of the catenary structures are shown in Figure 1b.

To achieve compact and spatiotemporally-controlled Bessel SPP beams, we elaborately designed the composite catenary structure according to our previous method [23], and the scanning electron microscope image is shown in Figure 1c. The composite catenary structure is formed by 4-column catenary aperture arrays (inclined by 10° along the *x*-axis and denoted as A1 to A4, respectively). The length of a single catenary aperture array is *L* = 8150 nm. The spacing between the catenary aperture arrays on the upper and lower sides is *d* = 500 nm. The catenary aperture arrays A1 and A3 are symmetrically distributed along the *x*-axis, so with catenary aperture arrays A2 and A4. It is important to note that the structural parameters can be further adjusted to match any other wavelength band [38]. Figure 1d shows a schematic diagram of the excitation of Bessel SPP beams by the composite catenary structure. As demonstrated in previous studies [17, 23], Bessel SPP beams can be formed by the stable interference of two inclined SPP plane waves. Thus, a single catenary aperture array in structure provides effective excitation and polarization-dependent active manipulation of SPP plane waves, as shown in Supplementary Information Figure S1. Meanwhile, the initial phase and wavefront of SPP are regulated by the arrangement design of multiple catenary aperture arrays in structure to effectively excite Bessel SPP beams [17]. Specifically, when the incident light illuminates the composite catenary structure, the SPPs launched from array A1 and array A2 (or arrays A3 and A4) will constructively interfere and form the Bessel SPP beams in the up region of the structure (or down region). It should be mentioned that when the incident light is obliquely incident on the excitation structure with an angle *γ*, it will cause the transmission path of the main lobe of the Bessel SPP beam to be shifted by an angle *β*, which satisfies the following formula [25, 26, 39]:

(2) $$\beta =\arcsin\left(\left(k_{0}\sin\left(\gamma \right)\cdot \cos\left(\theta \right)\right)/k_{\text{SPP}}\right)$$

where *k*_0_ is the wave vector of the incidence light, *k*_SPP_ is the wave vector of SPP, and *θ* is the tilt angle of the excitation structure. Specifically, due to the variation of the incident light angle, the transmission direction and initial phase distribution of the SPP excited by the catenary aperture array are affected (which can also be understood as the change of the tilt angle between the two SPPs when they were interfering), that results in the offset of the main lobe of Bessel SPP beam relative to the center of symmetry of the excitation structure. Considering the limitation of the experimental instrument of PEEM, the incident light is obliquely incident on the composite catenary structure with an angle of *γ* = 65° and excites Bessel SPP beams propagating obliquely upward and downward, respectively, as shown in Figure 1d. Besides, by changing the polarization of the incident light, the transmission path of the formed Bessel SPP beam can be actively controlled.

**Figure 2:**
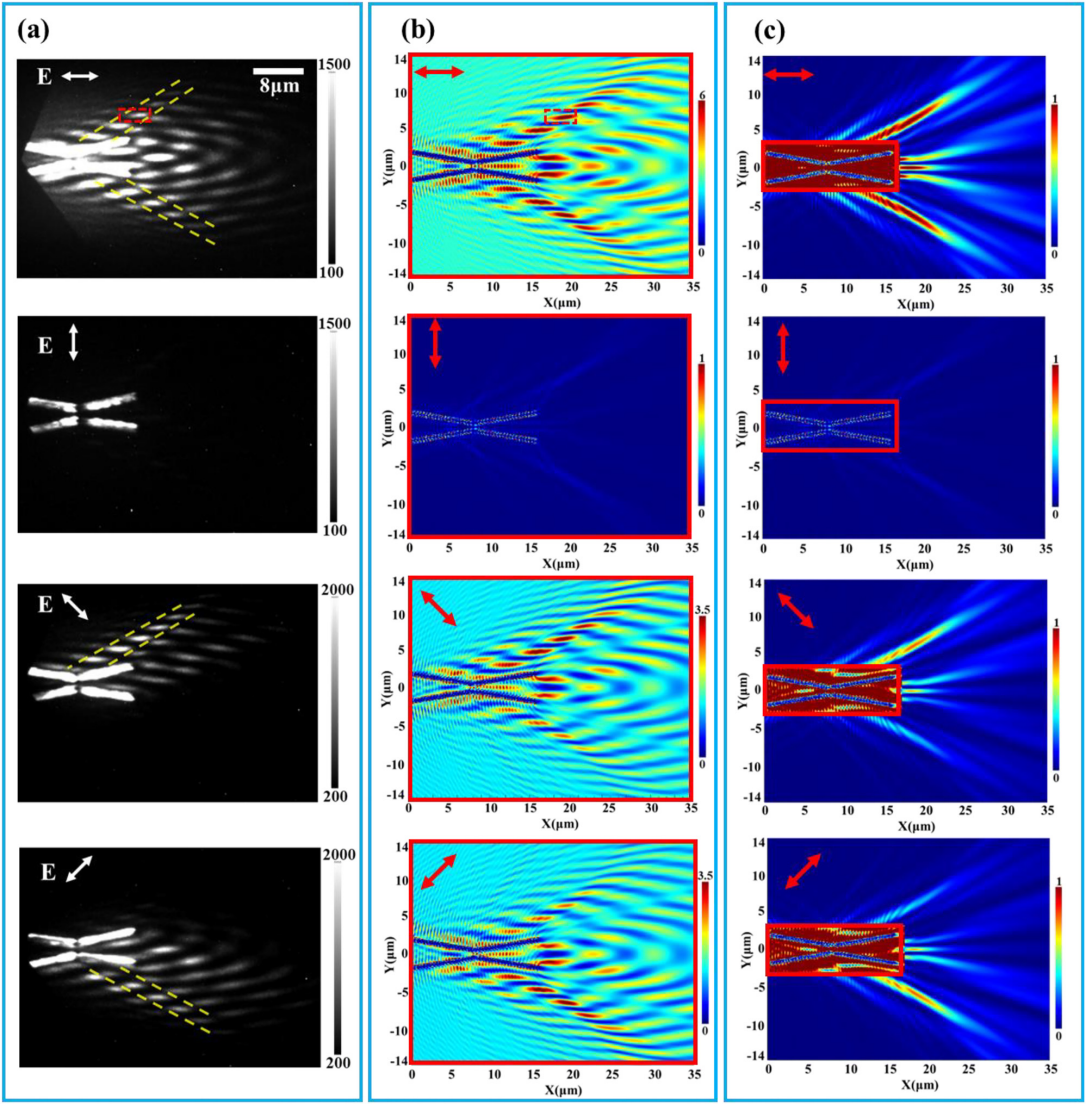
Polarization-dependent near-field distribution of Bessel SPP by PEEM experiment and FDTD simulation. (a) The PEEM images of the composite catenary structure following irradiation with different polarization states (*p* polarized, *s* polarized, 45° and −45° polarized). The yellow dashed lines mark the spatial distribution of the main lobes of the Bessel SPP beams. (b) The simulated near-field intensity distribution (E_z_^2^) of the total field. (c) The simulated near-field intensity distribution (E_z_^2^) of the pure SPP field. The red rectangles in (b, c) represent the region covered by the light.

As a prelude to the spatiotemporal control of Bessel SPP beams on the nano-femto scale, we first implement the excitation and spatial control of Bessel SPP beams by the composite catenary structure. We experimentally obtained the near-field image of SPP launched from the composite catenary structure under different linear polarization directions using PEEM. Figure 2a displays the PEEM images under irradiation of femtosecond pulses with different linear polarization angles (*p* polarized, *s* polarized, 45° polarized, and −45° polarized) (*λ* = 750 nm, 1.6 eV) on the composite catenary structure. Figure 2a shows the photoemission images under four different polarization states of a single incident light beam. Under p-polarized light illumination, as depicted in the upmost panel of Figure 2a, the SPPs will be effectively excited and launched symmetrically from the upper right and lower right of the structure. On the contrary, when excited by s-polarized light, as depicted in the second panel from the top of Figure 2a, only the localized surface plasmons (LSPs) signal on the structure can be observed. Furthermore, when the incident illumination light is turned to 45° (−45°) polarization, the SPPs excited in the composite catenary structure will be transmitted asymmetrically to the up (low) right of the structure, as depicted in the third (fourth) panel of Figure 2a. These phenomena confirm that the spatial control of SPP by the polarization direction of the incident light is realized in this catenary structure. Regarding the mechanism of polarization control of SPP launched from the composite catenary structure, it results from the competition between the excitation of LSP and SPP in the catenary apertures that affects the coupled excitation efficiency of SPP under different polarization excitations [40], thereby enabling polarization-controlled directional excitation of SPP beams under oblique incidence illumination (for more information, see Supplementary Information Figure S1). Note that Figure 2a shows a complex block-shaped pattern in each PEEM image. It originates from the interference between the Bessel SPP (called pure SPP interference, forming Bessel SPP beams) and the incident laser. Although the interference of the incident light field in the PEEM image cannot be directly excluded, the character of the main lobes of nondiffracting Bessel SPP beams can be recognized from the transmission trace as marked between the yellow dash lines in Figure 2a.

To further verify the imaging of Bessel SPP by PEEM, we performed the FDTD simulation to reproduce the experimental PEEM image patterns. The simulated near-field intensity distribution (*E*_*z*_^2^) with the incident light covering the whole total region of the image (the total-field case, analog to the PEEM image case) is displayed in Figure 2b. As shown in Figure 2b, the block-shape interference patterns can be clearly seen in the total-field light illumination case, which is well consistent with the experimental PEEM image in Figure 2a (as labeled by the marked red dotted rectangles in the upmost panels in Figure 2a and b). It should be mentioned that, due to the size and non-uniformity of the light spot in the experiment, the photoemission signal intensity at the center of the spot will be much stronger than that at the boundary of the spot in the PEEM images in Figure 2a.

In addition, the incident light that does not cover the detection region (as in the pure-SPP field case) for the composite catenary structure is shown in Figure 2c. It displays the simulated pure SPP interference near-field pattern in which the influence of the interference of the incident light and SPP is eliminated by restricting the illumination within the catenary structure region (within the red block region). It can be observed that the profile of the exciting SPP beam includes a central main lobe that propagates along a straight line with tiny divergence as well as several sub-lobes on both sides of the main lobe, which exhibits the apparent character of the Bessel SPP beam [24, 27, 41]. By comparing the simulated results with the PEEM images, we can confirm that the formed block patterns marked by the yellow dotted line in Figure 2a correspond to the main lobes of the Bessel SPP beams in Figure 2c. According to the PEEM images of the excited Bessel SPP beams, it can be observed that the main lobe of Bessel SPP beams propagates in a straight line maintaining and a tiny divergence angle (∼30 mrad) narrow waist width (∼1.6 μm).

Meanwhile, we quantitatively measured the shift angle (*β*) of the Bessel SPP beams of about 61° in PEEM images, which is in good agreement with the calculation of Equation (2). The self-healing properties of Bessel SPP beams formed under 65° oblique incidence excitation are verified by simulation (see Supplementary Information Figure S2). The extinction ratio R (defined as the ratio of the intensities of the SPPs excited on the upper and lower sides) of the composite catenary structure under 45° polarized light is about *R* = 3 in Figure 2c. The above results show that, under 65° oblique incident light illumination, the SPPs launched by the composite catenary structure will constructively interfere to form Bessel SPP beams propagating along the up-right and low-right directions of the structure. Meanwhile, by adjusting the polarization angle of the incident light, the directional excitation and switch of the Bessel SPP beams have been achieved.

**Figure 3:**
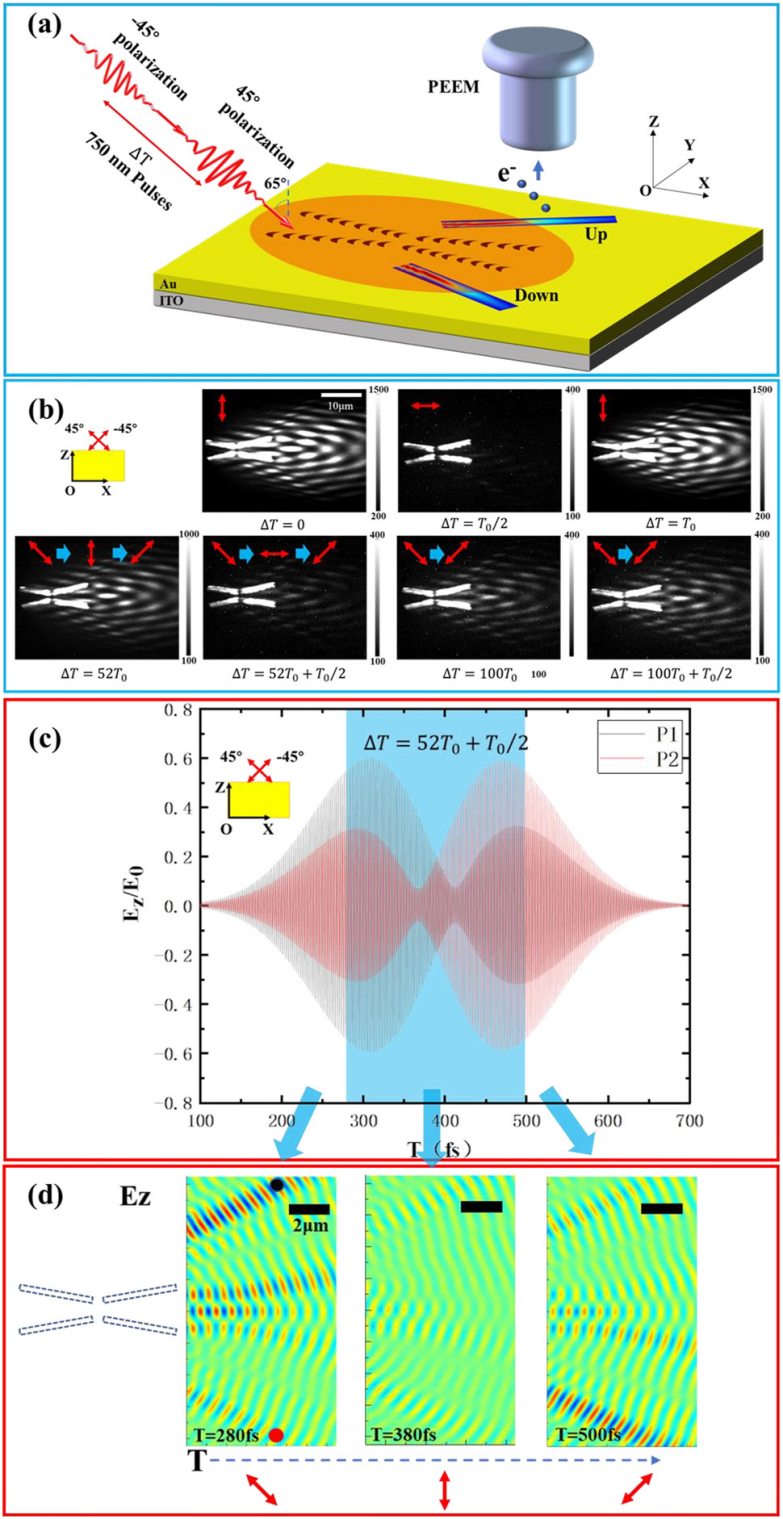
Ultrafast active control of Bessel SPP pulses. (a) Schematic diagram of the ultrafast control of Bessel SPP pulses by the composite catenary structure under the illumination of two time-delayed 750 nm pulses. (b) Time-resolved PEEM images of the composite catenary structure under different time delays of the two 45° polarized and −45° polarized pulses. The vector diagrams in (b) indicate the evolution of the different instantaneous polarization states. (c) Simulated temporal evolution of the near field of Bessel SPP beams at positions P1 and P2 as indicated in the (d). (d) The instantaneous near-field distribution of the Bessel SPP beams at different moments. Positions P1 (black dot) and P2 (red dot) are in the transmission path of the main lobe of Bessel SPP beams at *Y* = ±8 μm in (d). The vector diagram of the instantaneous polarization direction of the incident light is at the bottom in (d). The blue shaded area represents the time range of Bessel SPP beams excitation by the overlapped region of the two laser pulses. The black dotted frame in (d) indicates the position of the composite catenary structure.

The experimental results in Figure 2 demonstrated that the direction launching and switching of Bessel SPP beams on the composite catenary structure strongly depends on the polarization state of the incident light pulses. Based on this, it is foreseeable that ultrafast active control of the direction launching and switching of Bessel SPP pulses can be realized by manipulating the instantaneous polarization state of the incident light on the femtosecond time scale [42]. As shown in Figure 3a, for the two-beam PEEM experiment scheme, two 750 nm fs laser pulses with a relative time delay of Δ*T* were obliquely incident onto the composite catenary structure at 65° with respect to the surface normal. Then, the Bessel SPP pulses transmitted along the upper and lower sides of the structure are excited. Figure 3b displays the time-resolved PEEM images of the composite catenary structure under different time delays between 45° polarized and −45° polarized pulses. When the time delay is Δ*T* = 0, *T*_0_/2 and *T*_0_ (delay time is 0, 1.25 fs, and 2.5 fs, the optical period is *T*_0_ = 2.5 fs for the 750 nm carrier wave of the laser pulse), the transmission path case of Bessel SPP pulses in the up and down regions of the composite catenary structure turns from both-open (Δ*T* = 0) to both-closing (Δ*T* = *T*_0_/2), and then switching back to both-open (Δ*T* = *T*_0_). This phenomenon is attributed to the change in the polarization state of the incident light: under these time delays, the corresponding incident light polarization state after superposition is *p*, *s*, and *p* polarization. When the two pulses are completely separated in time, for example, longer than 100*T*_0_, the PEEM image is not related to the time delay between the two pulses anymore.

When Δ*T* = 52*T*_0_, delay time 130 fs (Δ*T* = 52*T*_0_ + *T*_0_/2, delay time 131.25 fs), the near field distribution in the PEEM image is consistent with the one at Δ*T* = 0 (Δ*T* = *T*_0_/2) except for a lower photoemission intensity. However, we should stress that the PEEM image at Δ*T* = 52*T*_0_ (Δ*T* = 52*T*_0_ + *T*_0_/2) actually includes an ultrafast switching of Bessel SPPs that is different from the case at Δ*T* = 0 (Δ*T* = *T*_0_/2). As the PEEM image corresponds to the integrating signal, it is hard to directly exhibit the ultrafast switching process occurring under different instantaneous polarization states from Figure 3b. The ultrafast switching process of Bessel SPP pulses can be revealed by further numerical simulations. Figure 3c shows the simulated temporal evolution of the near-field of Bessel SPP pulses at the P1 and P2 positions (denoted by black and red dots in Figure 3d) under the illumination of 45° and −45° polarized pulses with the time delay of 52*T*_0_ + *T*_0_/2 (delay time is 131.25 fs). The positions of P1 and P2 are selected at the main lobe of Bessel SPP pulses excited above and below the composite catenary structure. The blue-shaded area represents the temporally overlapped region of the two laser pulses. As time progresses, the Bessel SPP pulses intensity at P1 is initially stronger than that at P2, then the intensities at P1 and P2 gradually become weaker and tend to be the same (at nearly 400 fs), and it finally turns to be stronger at P2 than at P1. Note that, the intensity of the Bessel SPP pulses at the center of the evolution process (at around 390 fs when it exactly corresponds to *s* polarization for the overlapped field) in Figure 3c is not the weakest. This is due to the combination of the tilt of the catenary aperture array element in the composite catenary structure and the continuous variation of the overlapped laser field of two incident light pulses. To visualize the instantaneous change in the transmission path of the Bessel SPP pulses, we obtained the near-field distributions of the Bessel SPP pulses at three typical instantaneous times of *T* = 280 fs, 380 fs, and 500 fs, respectively, as shown in Figure 3d. It can be observed that the Bessel SPP pulses excited on the composite catenary structure will asymmetrically transmit upward at *T* = 280 fs and then transfer downward at *T* = 500 fs. And at time *T* = 380 fs, in the overlapping region of the two incident light pulses, the intensity of Bessel SPP at both sides of the catenary structure noticeably weakened. The above results show that during the entire electric field evolution process (at the hundreds of femtosecond scale), the transmission path of Bessel SPP pulses undergoes an ultrafast switching process from the upside to the downside position. This evolution process is consistent with the change of the instantaneous polarization state of the incident light, i.e., from 45° polarization to s-polarization, then to −45° polarization. Correspondingly, an ultrafast switching process of Bessel SPP pulses occurs when the time delay is 52*T*_0_. Besides, based on this control method, more potential control schemes for different ultrafast manipulation processes can be obtained (For more information, see Supplementary Information Figure S3).

**Figure 4:**
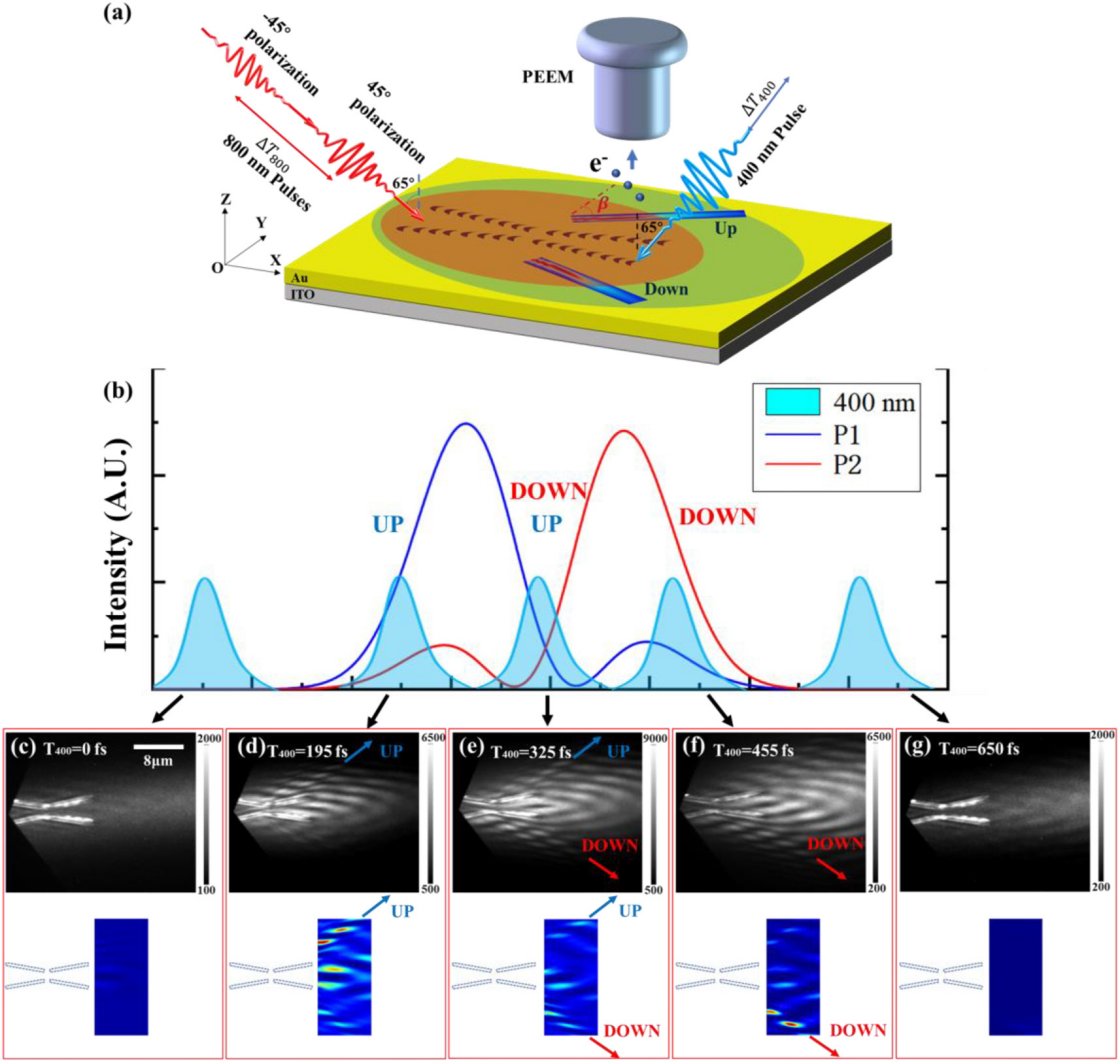
Revealing the ultrafast switching of Bessel SPP pulses by two-color three-beams PEEM. (a) Schematic diagram of the two-color three-beam PEEM experiment for obtaining the spatiotemporal information of Bessel SPP pulses. (b) The schematic demonstrates the ultrafast switching process of Bessel SPP pulses by the two-color three-beam PEEM images. Positions P1 and P2 are in the transmission path of the main lobe of Bessel SPP beams at *Y* = ±7 μm. (c–g) The two-color time-resolved PEEM images (upper panels) and corresponding FDTD simulation images (lower panels) of the composite catenary structure under different time delays between the pump and probe pulses. The time delay between two pump 800 nm pulses is keeping 48*T*_0_ + *T*_0_/2 (delay time is 129.43 fs, and the optical period is Δ*T* ≈ 2.667 fs for the 800 nm carrier wave of the laser pulse). The time delay between the probe pulse and the pump pulses increased progressively at 65 fs per step.

To experimentally reveal the ultrafast switching process of Bessel SPP pulses, a two-color three-beam TR-PEEM method is utilized [43–46]. The schematic diagram of the two-color three-beam PEEM experiment is shown in Figure 4a. In the experiment, two femtosecond laser pulses of 800 nm wavelength with a fixed relative time delay of ∆*T*_800_ = 129.43 fs were obliquely incident (at 65° relative to the surface normal) from the left side of the structure, which was used as pump pulses to achieve effective excitation and ultrafast control of the Bessel SPP pulses. Meanwhile, a 400 nm pulse, which results from the frequency doubling of 800 nm pulses and acts as the probe pulse to interact with the pump pulses and SPP, was obliquely incident at −65° from the right side of the structure with a time delay of Δ*T*_400_ relative to the pump pulses. The zero-time delay of Δ*T*_400_ is selected at the moment when the probe pulse initially encounters the preferential 45° pump pulse and causes the increment of the photoelectron signal. Then the photoelectrons induced by the laser pulses and Bessel SPP are captured by PEEM, enabling catching of the snapshot of the ultrafast switching process of Bessel SPP pulses on femtosecond time scales. As the resulting photoemission electron in the two-color PEEM case is dictated by the coherent interaction among the counter-propagating 400 nm probe pulse with the interference field arising from 800 nm pump pulses and the pump-initiated SPP field, the 400 nm pulse-swept PEEM image can directly reveal the distribution of Bessel SPP pulses in different instantaneous states (time window of about 140 fs corresponding to the 400 nm pulse duration) during the entire ultrafast switching process, as shown in Figure 4b. The opposite irradiation of a double-frequency (400 nm) probe pulse can improve the temporal resolution during spatiotemporal imaging by reducing the superposition time between the probe pulse and Bessel SPPs, and permit a better visualization of Bessel SPP pulses by avoiding the interference of 400 nm probe pulses and 800 nm pump pulses. Besides, it is known that higher nonlinear order of photoelectrons (corresponding to a longer wavelength of exciting femtosecond laser) will result in a more distinct brightness variation of PEEM image due to different degrees of two-color quantum channel opening [43, 47]. Accordingly, the wavelength of the pump pulse is redshifted from 750 nm to 800 nm for better visibility of the two-color PEEM image, which is strongly related to the nonlinear order of the photoelectrons [43, 45]. (For more information on two-color three-beam PEEM experimental images excited by 750 nm and 375 nm pulses, see Supplementary Information Figure S7). We should mention that although we used different excitation wavelength between one-color and two-color PEEM schemes, the underlying control mechanism and switching process was not affected.

Figure 4b depicts a schematic for the principle that reveals the ultrafast switching process of Bessel SPP pulses by the two-color PEEM. Figure 4c–g shows the two-color PEEM images and the corresponding FDTD simulation images at various relative time delays of 400 nm pulse (*T*_400_) related to pump pulses. It can be observed from Figure 4c that no Bessel SPP pulse pattern can be observed in the PEEM image at *T*_400_ = 0 fs time delay. This phenomenon shows that the PE signal generated by the pump-initiated Bessel SPP pulse alone will have a neglected contribution to the PEEM image when it does not interact with the 400 nm probe pulse due to the higher nonlinear order of the photoelectron. With the relative delay of the probe pulse increasing to *T*_400_ = 195 fs, as shown in Figure 4d, preferentially emitted Bessel SPP pulse from the upper side of the structure can be observed in the PEEM image, which is revealed through the opening of the two-color quantum channel formed by the joint contribution from the 400 nm probe and interference field resulting from the incident 800 nm light and SPP field [47]. At this time, the Bessel SPP pulse is dominated by the pump light excitation with an instantaneous polarization state of 45° and preferentially emitted to the upper right of the structure. When the relative delay is *T*_400_ = 325 fs, as shown in Figure 4e, Bessel SPP pulses appear to be emitted simultaneously from the upper and lower sides of the structure. The PEEM image at this moment corresponds to a distribution similar to the middle panel in Figure 3d. Furthermore, when the relative delay is *T*_400_ = 455 fs, as shown in Figure 4f, the preferred direction of the Bessel SPP pulse on the composite catenary structure is switched to the lower right side of the structure. This process corresponds to a distribution similar to the right panel in Figure 3d, which is dominated by the pump light excitation with an instantaneous polarization of −45°. Finally, when the relative delay is increased to *T*_400_ = 650 fs, the excited Bessel SPP pulse will be completely separated from the 400 nm probe pulse, and it vanishes again in the PEEM image of Figure 4g (For more two-color PEEM images with additional relative delays, see Supplementary Information Figure S6). Simulated PEEM images obtained by integrating the instantaneous electric field distribution signal in a specific time range are displayed in the below of Figure 4 (the detailed simulation setup is shown in the Supplementary Information). A good consistency between the experimental and simulated PEEM images confirms the reliability of these experimental results. The near-field distributions shown in the experimental two-color PEEM images directly demonstrate that the ultrafast manipulation of Bessel SPP pulses has been realized in the composite catenary structure. We believe the current experiment provides an effective and feasible means for direct ultrahigh spatiotemporal mapping of the ultrafast switching of nondiffracting SPP pulses, as well as guidance for the practical application of ultrafast nondiffracting SPP pulses.

## 3 Conclusions

In summary, we proposed an active control scheme of Bessel SPP beams based on the composite catenary structure. The results show that, by changing the instantaneous polarization states of incident light that is achieved via adjusting the time delay of two orthogonally polarized incident laser pulses, femtosecond timescale switching of the Bessel SPP pulses had been realized. More importantly, by adding an additional opposite-irradiation frequency doubling pulse, we experimentally mapped the femtosecond scale switching process of the Bessel SPP pulses using a two-color three-beam PEEM. Both of the results are well reproduced by FDTD simulations. Our work establishes a new experimental path for spatiotemporal controlling of nondiffracting Bessel SPP and has potential for applications in integrated nanocircuitry [10, 48] and ultrafast photonic information processing [49]. Moreover, the two-color three-beam PEEM presented here offers a paradigm candidate for experimentally mapping the ultrafast manipulation of nondiffracting SPP pulses [30] and can extend the scope of TR-PEEM research from imaging of the plasmonic fields in the visible regime to near-infrared regime, or from metal samples to monolayer semimetals/semiconductors or topological insulators materials [33].

## 4 Experimental section

### 4.1 Sample fabrication

The catenary aperture array consisting of twenty elements with a spaced period of 400 nm is fabricated in a gold film with a thickness of 120 nm by focused ion beam lithography, and the SEM images of the catenary structures are shown in Figure 1b and c. The catenary aperture goes all the way through the gold film with a thickness of approximately 120 nm. The thickness of the gold film (120 nm) is much larger than the skin depth of Au [50], which ensures sufficient coupling of the SPP field only at the Au/vacuum interface. The gold film was deposited on a SiO_2_ substrate coated with an approximately 180 nm thick indium tin oxide (ITO) layer via sputtering. A ∼3 nm Ti adhesion layer was deposited between the gold film and ITO.

### 4.2 Experiment measurement

A femtosecond Ti-Sapphire laser oscillator (Coherent, Mira 900) with a repetition rate of around 76 MHz and a bandwidth of 6 nm is used to excite the SPP. The pulse duration is 130 fs and the output wavelength of the laser can be tuned within 700–900 nm (1.77–1.38 eV). The laser power used in the experiment was 100 mW. The photoemission electron microscope (PEEM, Focus GmbH, Hünstetten, Germany) was employed to record the multiphoton photoemission from a superposition of SPP and laser field. The incident lasers in the experiment are focused on the sample surface with an incident angle of 65°. Under this condition, elliptically-shaped focused laser spots are observed with the major and minor axes of 60 μm and 30 μm, respectively. For time-resolved PEEM experiments, a pulse is split in a stable Mach–Zehnder interferometer with a tunable time delay. Independent polarization and power control of the laser pulse in each arm can be achieved via half-wave plates and continuously adjustable attenuation plates. For two-color three-beam time-resolved PEEM experiments, an additional third laser pulse beam was frequency-doubled into 400 nm (probe pulse) through a β-BaBO3 (BBO) crystal and irradiated on the sample surface with oblique incidence (−65°) opposite to the sample surface. The delay of the 400 nm probe laser relative to the two 800 nm laser pulses is independently controlled by another translation stage.

### 4.3 Numerical simulation

The FDTD calculations are performed with the FDTD Solutions commercial software (Lumerical, Ltd). In order to allow monitoring of either the total field (including light and SPP) or pure SPP interference field only (without incident light), a total field scattered field source (TFSF) is employed in the calculations. And the instantaneous field distribution of the SPP near field was captured by the time dimension monitor. The incident light parameters and structure of the sample in the simulation are consistent with the experiment. The dielectric constant of gold was fitted to experimental data from Johnson and Christy [51]. The ITO-covered glass substrate was assumed to behave as a dielectric with an average refractive index of *n* = 1.55. The surrounding medium is a vacuum. The mesh size nearby the sample was 7 nm × 7 nm × 5 nm, and the perfectly matched layer (PML) boundary condition is adopted to reduce artificial reflections from the boundaries. A convergence study had performed, and the error was within the acceptable limit.

## Supplementary Material

Supplementary Material Details
