# Supplementary material for: High spatiotemporal resolved imaging of ultrafast control of nondiffracting surface plasmon polaritons
Source: Nanophotonics. 2023 May 16;12(12):2121–31. doi: 10.1515/nanoph-2023-0074 (PMC11501457; doi:10.1515/nanoph-2023-0074)
Supplement: Supplementary file 1 — Supplementary Material Details [file j_nanoph-2023-0074_suppl.docx]

**Supplementary Information**

**High spatiotemporal resolved imaging of ultrafast control of nondiffracting surface plasmon polaritons**

*Hanmin Hu, Boyu Ji*, Lun Wang, Peng Lang, Yang Xu, Zhenlong Zhao, Xiaowei Song and Jingquan Lin**

**1. The mechanistic explanation of polarization-controlled SPP**

**Figure S1.** (a, b) The PEEM images of the single catenary aperture array following irradiation with different polarization states (45° and -45° polarized). (c, d) FDTD simulated near-field intensity maps (${E_{z}}^{2}$) of the single catenary aperture array with different polarization states (45° and -45° polarized). Surface plasmon field enhancement line profiles (e) and (f) were calculated at the positions indicated by solid red lines in (c) and (d), respectively. (g) FDTD simulated near-field intensity maps (${E_{z}}^{2}$) of the opposite single catenary aperture array. Surface plasmon field enhancement line profiles (h) are calculated at the positions indicated by solid red lines in (g). The catenary aperture element position is marked by two dashed arrows at the top center of (e), (f), and (h).

Figures S1a and S1b display the PEEM images of the single catenary aperture array illuminated by the 65° oblique incidence light with 45° and -45° polarization. Under 65° oblique incident light illumination, the single catenary aperture array will excite and launch SPPs. The asymmetric surface field distributions can be clearly observed for the two different pump polarization. And the same phenomenon can be seen in the FDTD simulation, as shown in Figures S1c and S1d. Surface field intensity line profiles are displayed in Figures S1e and S1f. When the field polarization is rotated to 45°, the intensity line profile shows that the SPP field on the flat Au surface is stronger on the left side of the catenary aperture when compared with the right side (Figure S1e). When the field polarization is rotated -45°, the left/right distribution is reversed (Figure S1f). In addition, there are field intensity spikes at both edges of the trench, attributed to field enhancement at the sharp apex of the catenary aperture edge. It has been investigated that when the pump is 45° or -45° polarized, more light can couple to the localized plasmon field, and the generated SPP field is comparatively weak^[47]^. Once again, the same phenomenon can be clearly observed for the opposite single catenary aperture array under 45° polarization, as shown in Figure S1g and S1h. Thus, the single catenary aperture array can polarization-control SPP under 65° and -65° oblique incidence illumination.

**2. FDTD simulation of the self-healing properties**

**Figure S2.** The simulated near-field intensity distribution ($E_{z}^{2}$) of the Bessel SPP beam generated by the composite catenary structure after facing an Au cylindrical obstacle. The white circle in the Figure is the marked cylindrical obstacle with a diameter of 300𝑛𝑚, and a height of 70nm.

To confirm the self-healing property of Bessel SPP beams excited by the composite catenary structure under 65° oblique incidence illumination, we introduce a defect in the forward path of the main lobe of the Bessel SPP beam in the form of a cylindrical gold obstacle with a diameter of 200 nm and a height of 70 nm on the gold film. As illustrated in Figure S2, after passing through a cylindrical obstacle, the excited Bessel SPP beams can still propagate in a straight line. More importantly, the intensity distribution is essentially the same as the one without the obstacle in Figure 3d. Thus, the Bessel SPP beams excited by the composite catenary structure keep the self-healing property under 65° oblique incidence illumination.

**3. The ultrafast control scheme based on the p-polarized and s-polarized pulses**

**Figure S3.** (a) Schematic diagram of the ultrafast control of Bessel SPP pulses by the composite catenary structure under two 750 nm p-polarized and s-polarized pulses. (b) Time-resolved PEEM images of the composite catenary structure under different time delays of the two P-polarized and S-polarized pulses. The vector diagrams in (b) indicate the evolution of the instantaneous polarization states of the overlapped field of two incident laser pulses. (c) Simulated temporal evolution of the near field of Bessel SPP beams at positions P1 and P2 as indicated in the (d). (d) The instantaneous near-field distribution of the Bessel SPP beams at different moments. Positions P1 (black dot) and P2 (red dot) are in the transmission path of the main lobe of Bessel SPP beams at Y=±8 μm. The vector diagram of the instantaneous polarization direction of the incident light is denoted at the bottom of (d). The blue shaded area represents the time range of Bessel SPP beams excitation by the overlapped laser field of the two pulses. The black dotted frame in (d) indicates the position of the composite catenary structure.

Drawing on the control scheme introduced in Figure 3, we further designed a different ultrafast control scheme based on the p-polarized and s-polarized incident light pulse pairs, as shown in Figure S3a. Figure S3b displays the time-resolved PEEM images of the composite catenary structure under different time delays between p-polarized and s-polarized pulses. When the time delay is ΔT=-T_0_/2, 0 and T_0_/2 (the real time delay is -1.25 fs, 0 fs, and 1.25 fs, respectively, as the optical period is T_0_=2.5 fs for the 750 nm laser pulse), the transmission path of Bessel SPP pulses turns from down (ΔT=-1.25 fs) to up (ΔT=0), and then switching back to down (ΔT=1.25 fs). This phenomenon is attributed to the change of the polarization state of the incident light: under these time delays, the corresponding polarization state of the incident light after superposition is -45°, 45°, and -45° polarization, respectively. When the two pulses are completely separated in time, for example, longer than 100T_0_, the PEEM image is not related to the time delay between the two pulses anymore.

Figure S3c shows the simulated temporal evolution of the near-field of Bessel SPP pulses at the P1 and P2 positions (denoted by black and red dots in Figure S4c) under the illumination of p and s polarized pulses with the time delay of 52T_0_ (delay time is 130 fs). At the beginning, the Bessel SPP pulse intensity at P1 and P2 are both strong. At nearly 300 fs, the intensities at P2 gradually decrease and become weaker than P1, and it finally both decreases and becomes the same for P1 and P2 at nearly 550 fs. To visualize the instantaneous change in the transmission path of the Bessel SPP pulses, we obtained the near-field distribution snapshots of the Bessel SPP pulses at three instantaneous times of T=280 fs, 420 fs, and 520 fs, respectively, as shown in Figure S3d. It can be observed that the Bessel SPP pulses excited on the composite catenary structure will symmetrically transmit up and down at T=280 fs. And at time T=420 fs, in the overlapping region of the two incident light pulses, the Bessel SPP pulses will asymmetrically transmit upward. At time T=520 fs, the intensity of Bessel SPP at both sides of the catenary structure noticeably weakened. Correspondingly, an ultrafast switching process of Bessel SPP pulses occurs when the time delay is 52T_0_+T_0_/2.

**4. The setup of the three-beam two-color PEEM experiment**

**Figure S4.** Schematic diagram of the optical path for three-beam two-color TR-PEEM experiment.

The three-beam two-color TR-PEEM experimental optical path diagram used in the experiment is shown in Figure S4. In the experiment, two 800 nm pump pulses with polarizations of 45° and -45° are 65° oblique incident on the sample surface, and the time delay of ${{48T}_{0}+T}_{0}/2$ is fixed between these two pulses. The 400 nm probe pulse is irradiated on the sample surface with oblique incidence (-65°) opposite to the sample surface. The delay of the 400 nm probe laser relative to the two 800 nm laser pulses is controlled by another translation platform.

**Figure S5.** (a) Simulated temporal evolution of the near field of Bessel SPP pulses at positions P1 and P2 (excluding the incident light pulse). (b) Simulated temporal evolution of the near field of total interference field at positions P1 and P2 (including the incident pump light pulse). Positions P1 (black dot) and P2 (red dot) are in the transmission path of the main lobe of Bessel SPP beams at Y=±7 μm.

Under this time delay between two pump pulses, the Bessel SPP pulse generated by two pump pulses will undergo an ultrafast spatiotemporal manipulation process similar to that in Figure 4b, and the specific SPP signal switching evolution is shown in Figure S5a. The process of the ultrafast manipulation of Bessel SPP pulses by the composite catenary structure under laser pulse illumination can be roughly divided into the following three steps: Step 1, Bessel SPP pulses are excited by the first laser pulse and preferentially transmitted to P1, and the corresponding time range is 0-280 fs; Step 2, Bessel SPP pulses are excited by the overlapping part of the two laser pulses with the variation of transmission direction, and the corresponding time range is 280-500 fs; Step 3, Bessel SPP pulses are excited by the second half of the second beam and preferentially transmitted to P2 after 500 fs.

It should be mentioned that there is a certain delay in the relative time between the incident light pulse and the corresponding excited SPP reaching the P point, and the polarization of the incident light pulse changes with the time delay. Thus, as shown in Figure S5b, the switching time of the total interference field signal (including the incident pump light pulse) is earlier than that of Bessel SPP pulses. Meanwhile, the switching time difference decreases as the distance from the structure decreases. Based on the above reasons, deduce the intensity of SPPs near the arrow is similar, while the intensity of SPPs near the structure will have some differences in the PEEM image, as shown in Figure 4e.

Meanwhile, the FDTD simulated PEEM images in Figures 4(c-g) are obtained by integrating the instantaneous electric field distribution signal in a specific time range. The specific integration time range is mainly determined by the pulse width of the 400 nm probe pulse. In addition, the quantum channel opening degree and the total interference field intensity will also affect the photoelectron signal in the PEEM image. The final integration time range of the simulated PEEM image will be constrained in the time region where two 800 nm pump pulses destructively interfere, which is similar to the case of the lower panel in Figure 4e.

**Figure S6.** (a-l) Three-beams Time-resolved PEEM images of the composite catenary structure under different time delays were captured using two 800 nm pulses and another 400 nm pulse.

Besides, Figures S6(a-l) display the two-color PEEM images with additional relative delays. It can be observed the complete ultrafast switching process of Bessel SPP pulses in hundreds of femtoseconds. And the result of PEEM images is in good agreement with the simulated ultrafast switching process in Figure S5.

We utilized the pump light sources with different wavelengths in the one-color two-beam and the two-color three-beam PEEM experiment. To further reveal this case, we presented the PEEM images under the excitation of 750 nm+375 nm (shown in Figure S7a) and 800 nm+400 nm (shown in Figure S7b), respectively. It can be seen from Figure S7a, with extending the time delay of 375 nm laser pulse from T_375_=260 fs to 520 fs, the brightness variation of the upper and lower fringes are not obvious. Especially, the fringes launched to the upper and lower directions are visible throughout the time-resolved PEEM images at different time delays for 375 nm laser pulse. In addition, the brightness of the region beyond the fringes of Bessel SPP pulses in the PEEM images is also quite strong, which undoubtedly increases the background intensity and reduces the visibility of the image. Figure S7b shows the time-resolved PEEM image under the excitation of 800 nm+400 nm pulses. It can be seen that, at the beginning (T_400_=260 fs), only the fringes propagated upward can be observed. With the increase of pulse delay for the 400 nm laser, the upper fringes gradually disappeared, accompanied by the appearance of the downward fringes (at roughly T_400_=390 fs).

A better visualization of the PEEM images under the illumination of 800 nm+400 nm laser pulse pair than the case of 750 nm+375 nm laser pair results from the fact that the order of photoemission electrons excited by 750 nm is lower than the one excited by 800 nm. It is known that the higher nonlinear order of the photoemission electrons induced by the fundamental frequency laser excitation, the more obvious photoemission increase for the corresponding two-color (fundamental frequency laser combining with frequency doubling laser) PEEM image[1]. Accordingly, the brightness enhancement of the nondiffracting SPP beam by the excitation of 750 nm+375 nm assembly is weaker than the one of 800 nm +400 nm assembly. In addition, due to the higher photon energy of 375 nm light compared with 400 nm light, the illumination of 375 nm laser led to excessively strong photoelectron emission yield from the surface of the sample (equivalent to an excessively stronger background) as shown in Figure S7a. In short, the visibility of the two-color three-beam PEEM images with 800 nm+400 nm assembly is significantly better than the one with 750 nm+375 nm assembly. Based on the above reasons, we chose the wavelength pair of 800 nm+400 nm in the two-color three-beam excitation to obtain a better visualization. We should mention that although we used different excitation wavelength between one-color and two-color PEEM schemes, the underlying control mechanism and switching process was not affected.

**Figure S7.** Two-color three-beam PEEM images of the composite catenary structure excited by two 750 nm pulses and a 375 nm pulse (a) and by two 800 nm pulses and a 400 nm pulse (b).

**Reference：**

[1] B. Ji, X. Song, Y. Dou, H. Tao, X. Gao, Z. Hao, J. Lin, Two-color multiphoton emission for comprehensive reveal of ultrafast plasmonic field distribution, New J. Phys. 20 (2018) 073031. https://doi.org/10.1088/1367-2630/aad145.
